# The Association of Primary Anesthesia Type With Postoperative Transfusion in Anemic Patients Undergoing Primary Total Joint Arthroplasty

**DOI:** 10.7759/cureus.24496

**Published:** 2022-04-26

**Authors:** Joseph Kim, Brian P Curran, Austin L Du, Rodney A Gabriel

**Affiliations:** 1 Anesthesiology, Kaiser Permanente, Fontana, USA; 2 Anesthesiology, University of California San Diego, La Jolla, USA

**Keywords:** total hip arthroplasty, total knee arthroplasty, general anesthesia, neuraxial anesthesia, anemia

## Abstract

Background and objective

A high rate of preoperative anemia has been observed in patients undergoing knee and hip arthroplasty. The type of anesthesia that patients receive may play a role in preventing or minimizing adverse outcomes in these patients. In this study, we aimed to examine the complication rates in patients with severe anemia undergoing this surgery. In addition, we explore whether neuraxial anesthesia is associated with better outcomes compared to general anesthesia.

Methods

The American College of Surgeons National Surgical Quality Improvement Program (ACS NSQIP) registry was used to extract data related to patients who underwent total hip or knee arthroplasty from 2014 to 2016. Only those patients with a hematocrit level <30% were included, and they were classified into two groups based on primary anesthesia type received: neuraxial versus general anesthesia. The primary outcome of interest was postoperative transfusion. Secondary outcomes included 30-day hospital readmission and postoperative complications. Multivariable logistic regression was used to model primary anesthesia type to outcomes while controlling for various confounders. The odds ratio (OR) and their 95% confidence intervals (CI) were reported.

Results

There were 1,723 patients with severe anemia included in our analysis, of which 41.2% received neuraxial anesthesia. Among patients that received neuraxial versus general anesthesia, 170 (31.08%) and 486 (41.33%), respectively, received a postoperative blood transfusion (p<0.001). On multivariable regression analysis, neuraxial anesthesia was associated with 40% decreased odds of postoperative transfusion (OR: 0.63, 95% CI: 0.51-0.79, p<0.0001), but it was not associated with any other outcomes.

Conclusion

Neuraxial anesthesia can reduce the risk of postoperative transfusion in severely anemic patients undergoing total joint arthroplasty (TJA), ultimately leading to reduced discomfort, hospital expenditure, and adverse outcomes.

## Introduction

Total knee and hip arthroplasties are two of the most common orthopedic procedures performed in the United States, providing pain relief, functional recovery, and improved quality of life [1]. Currently, the annual growth rate for arthroplasties is 8% in the United States, with a projected total of four million cases of the knee and hip arthroplasties to be performed annually by the year 2030 [2,3]. Despite the growing demand and considerable advances in total joint arthroplasty (TJA), adverse outcomes still occur. Anemia, a common yet significant risk factor, increases the likelihood of patients having postoperative complications [4].

Prospective and retrospective studies have confirmed that pre- and postoperative anemia is highly prevalent in TJA patients [5]. Among patients undergoing total hip or knee arthroplasty and hip fracture surgery, preoperative anemia was present in about 24% and 44%, respectively. Postoperative anemia is more prevalent at rates of about 51% and 87%, respectively. Blood transfusions were also common in both procedures, occurring in almost half of the cases [5]. These high rates of anemia contribute to postoperative infections, poorer physical functioning, delayed recovery, increased length of hospital stay, higher mortality rates, and an increased burden on healthcare expenditure [4]. Cases of preoperative anemia are most commonly treated with iron, erythropoietin, and cell savage, which decreases the need for allogeneic blood transfusions while improving patient outcomes.

A prospective observational study conducted in northern England and Wales in 2000 and 2014 showed that roughly 41% of blood transfusions were done for surgical patients, with total hip and knee arthroplasty and hip-fracture repair being the leading indications for surgical patients, accounting for roughly 8% of all transfused units [6,7]. In addition, the leading demographic indicated for blood transfusions in multiple countries, unsurprisingly, were older patients [8]. With a shortage of blood products combined with a rapidly aging United States population and the risk of transfusion-related complications, the need to reduce blood product utilization is of paramount importance. Early detection and management of preoperative anemia before an elective surgery have been shown to reduce the need for blood transfusions [9].

Although detection of perioperative anemia is of primary importance, anesthesia may play a role in preventing or minimizing adverse outcomes in patients with severe anemia, which we define as a hematocrit level <30%, based on previous studies [10]. In particular, neuraxial anesthesia as an alternative to general anesthesia has been associated with decreased transfusion and postoperative complication risk following TJA [3]. The purpose of this study is to conduct a retrospective analysis to report the rate of postoperative transfusion and complication rates, by comparing general and neuraxial anesthesia outcomes in patients with perioperative anemia. Specifically, we aim to report the transfusion rates, 30-day readmission, and 30-day postoperative complications in patients with severe anemia, defined as a preoperative hematocrit level of less than 30% [11]. In addition, we analyze whether neuraxial anesthesia is associated with better outcomes compared to general anesthesia in this specific population.

## Materials and methods

As this study was not considered human subjects research and data were extracted from a public de-identified database, our institutional review board (Human Research Protections Program) waived the requirement for consent. The American College of Surgeons National Surgical Quality Improvement Program (ACS NSQIP) registry was used to extract all patient records. The ACS NSQIP is a nationally validated surgical outcome registry used extensively to improve health outcomes.

The inclusion criteria were as follows: patients aged ≥18 years who underwent primary TJA from 2014 to 2016 and who were classified as severely anemic (preoperative hematocrit level <30%). We identified the cases based on the current procedural terminology codes of 27447 (total knee arthroplasty) and 27130 (total hip arthroplasty). The primary outcome of interest was postoperative transfusions. Secondary outcomes of interest included 30-day readmissions and 30-day postoperative complications, which included the following: reintubations, pneumonia, pulmonary embolism, urinary tract infection, stroke, myocardial infarction, or deep vein thrombosis. The primary exposure variable was the primary anesthesia type (general anesthesia vs. neuraxial anesthesia).

The additional covariates collected were as follows: use of general anesthesia as the primary anesthetic (vs. neuraxial anesthesia), sex, age (classified into <65 years, ≥65 to <80 years, and ≥80 years), race, surgical procedure, preoperative transfusion received, wound infection at the time of operation, diabetes mellitus, preoperative dyspnea, severe chronic obstructive pulmonary disease, hypertension, preoperative steroid use, and preoperative bleeding disorder. We excluded all cases with missing data for anesthesia type and those with unknown preoperative hematocrit levels. We only included patients with hematocrit levels of less than 30%. None of the other covariates had any missing data.

Statistical analysis

Statistical analysis was performed using RStudio, a software environment for statistical computing (R version 1.3.1093). On unadjusted analysis, to measure differences in the outcomes and patient characteristics between the general anesthesia and neuraxial anesthesia cohorts, we used the chi-squared tests. We then performed a multivariable logistic regression analysis to model the association of primary anesthesia type (neuraxial vs. general anesthesia) with the need for postoperative transfusion. Our multivariable model controlled for sex, age, surgical procedure, preoperative transfusion requirements, wound infection at the time of operation, diabetes mellitus, preoperative dyspnea, severe chronic obstructive pulmonary disease, hypertension, preoperative steroid use, and bleeding disorder. The odds ratio (OR) and its 95% confidence intervals (CI) were then reported for each covariate. This statistical approach was repeated for secondary outcomes occurring within 30 days postoperatively: reintubation, pneumonia, pulmonary embolism, urinary tract infection, readmission, myocardial infarction, deep vein thrombosis, and stroke. Because we were looking at nine different outcomes, we chose a p-value of less than 0.0055 as statistically significant after applying the Bonferroni correction.

## Results

Data extraction from the ACS NSQIP yielded records of 231,899 adult patients who underwent primary total hip or knee arthroplasty; of them, 1,723 (0.7%) were classified as severely anemic. Among the patients who were severely anemic, 866 (50.3%) underwent total hip arthroplasty, 857 (49.7%) underwent total knee arthroplasty, 547 (31.7%) received neuraxial anesthesia as their primary anesthetic, 528 (30.6%) were men, and 723 (42.0%) were aged between ≥65 and <80 years. Significant differences in wound infection at the time of operation (p=0.02) and history of a prior bleeding disorder (p<0.001) were observed between patients receiving general and neuraxial anesthesia. Only four (0.70%) patients receiving neuraxial anesthesia had a wound infection at the time of operation compared to 31 (2.60%) patients receiving general anesthesia (p=0.015). Nineteen (3.50%) patients receiving neuraxial anesthesia had a history of a prior bleeding disorder compared to 105 (8.90%) patients receiving general anesthesia (p<0.001) (Table 1).

**Table 1:** Characteristics of Patients Undergoing Primary Total Joint Arthroplasty With Severe Anemia (Hematocrit <30%)

Patient Characteristics	General Anesthesia		Neuraxial Anesthesia		P-value
	N	%	N	%	
Total	1,176		547		
Male Sex (%)	350	29.80%	178	32.50%	0.26
Race (%)					<0.001
American Indian or Alaska Native	10	0.90%	2	0.40%	
Asian	16	1.40%	16	2.90%	
Black or African American	217	18.50%	88	16.10%	
Native Hawaiian or Pacific Islander	9	0.80%	3	0.50%	
Unknown/Not Reported	82	7%	88	16.10%	
White	842	71.60%	350	64%	
Age (%)					0.03
<65 years	478	40.60%	200	36.60%	
>65 and <80 years	498	42.30%	225	41.10%	
>80 years	200	17%	122	22.30%	
Surgical Procedure					0.002
Total Knee Arthroplasty	555	47.20%	302	55.20%	
Total Hip Arthroplasty	621	52.80%	245	44.80%	
Preoperative Transfusion Received	81	6.90%	27	4.90%	0.15
Wound Infection at the Time of Operation	31	2.60%	4	0.70%	0.02
Severe Chronic Obstructive Pulmonary Disease	86	7.30%	30	5.50%	0.19
Steroid Use for Chronic Condition	98	8.30%	48	8.80%	0.83
Prior Bleeding Disorder	105	8.90%	19	3.50%	<0.001
Diabetes					0.43
Insulin-dependent	113	9.60%	42	7.70%	
No Diabetes	900	76.50%	427	78.10%	
Non-Insulin-dependent Diabetes	163	13.90%	78	14.30%	
Hypertension Requiring Medication	781	66.40%	374	68.40%	0.45
Dyspnea					0.85
At Rest	7	0.60%	3	0.50%	
Moderate Exertion	77	6.50%	32	5.90%	
No	1,092	92.90%	512	93.60%	

Among patients who were severely anemic, 656 (38.07%) required a blood transfusion postoperatively, 125 (7.25%) experienced 30-day readmission, and 19 (1.10%) required a reintubation. Among patients who received neuraxial versus general anesthesia, 170 (31.08%) and 486 (41.33%), respectively, received a postoperative blood transfusion (p<0.001) (Table 2). There were no statistically significant differences in other outcomes between the cohorts.

**Table 2:** Comparison of Postoperative Outcomes in General vs. Neuraxial Anesthesia for Patients With Severe Anemia (Hematocrit <30%) Undergoing Total Joint Arthroplasty *Statistically significant with p<0.0055

30-day Postoperative Outcomes	Entire Population		General Anesthesia		Neuraxial Anesthesia		P-value
	N	%	N	%	N	%	
Total	1,723		1,176		547		
Postoperative Transfusion	656	38.07%	486	41.33%	170	31.08%	<0.001*
Reintubation	19	1.10%	17	1.45%	2	0.37%	0.08
Postoperative Pneumonia	30	1.74%	22	1.87%	8	1.46%	0.68
Postoperative Pulmonary Embolism	15	0.87%	8	0.68%	7	1.28%	0.33
Postoperative Urinary Tract Infection	27	1.57%	19	1.62%	8	1.46%	0.97
Readmission	125	7.25%	85	7.23%	40	7.31%	0.41
Postoperative Myocardial Infarction	7	0.41%	3	0.26%	4	0.73%	0.29
Postoperative Deep Vein Thrombosis	19	1.10%	14	1.19%	5	0.91%	0.87
Postoperative Stroke	1	0.06%	1	0.09%	0	0.00%	0.99

In multivariable logistic regression analysis, we tested the association of primary anesthesia type with each outcome of interest. Those receiving neuraxial anesthesia had lower odds of postoperative transfusion compared to those receiving general anesthesia (OR: 0.63; 95% CI: 0.51-0.79; p<0.001) (Table 3). There was no statistically significant association between primary anesthesia type and secondary outcomes, otherwise.

**Table 3:** Results of Multivariable Logistic Regression for Neuraxial vs. General Anesthesia for Postoperative Outcomes *Statistically significant with p<0.0055 The model controlled for sex, age, race, surgical procedure, preoperative transfusion requirements, diabetes, preoperative dyspnea, severe chronic obstructive pulmonary disease, wound infection at the time of surgery, hypertension, preoperative steroid use, and bleeding disorder history CI: confidence interval

30-day Postoperative Outcomes	Odds Ratio (95% CI)	P-value
Postoperative Transfusion	0.63 (0.51-0.79)	<0.001*
Reintubation	0.21 (0.04-1.09)	0.06
Postoperative Pneumonia	1.11 (0.46-2.72)	0.81
Postoperative Pulmonary Embolism	2.55 (0.84-7.78)	0.09
Postoperative Urinary Tract Infection	0.86 (0.36-2.06)	0.73
Readmission	1.09 (0.73-1.65)	0.65
Postoperative Myocardial Infarction	0.18 (0.03-0.95)	0.04
Postoperative Deep Vein Thrombosis	0.96 (0.11-8.18)	0.97
Postoperative Stroke	0 (2.40e-282 - 1.68e+271)	0.97

## Discussion

In this study, we demonstrated that there was a low rate of various 30-day postoperative medical complications following TJA in patients with severe anemia. Unsurprisingly, there was a high rate (approximately 40%) of postoperative transfusions in this population. The use of neuraxial anesthesia was associated with an approximately 40% decreased odds of postoperative transfusions compared to the use of general anesthesia. This suggests that when patients with severe anemia (<30% hematocrit) undergo TJA, neuraxial anesthesia is the optimal choice for reducing the odds of postoperative transfusions. The odds of other complications, however, were unchanged.

Compared to patients with severe preoperative anemia, the general population undergoing TJA had lower rates of postoperative complications, including the need for transfusions, reintubations, pneumonia, pulmonary embolism, urinary tract infection, myocardial infarction, deep vein thrombosis, and stroke [12]. Specifically, the overall transfusion rate in the general population undergoing arthroplasties was 9% and 4% after total knee and total hip arthroplasty, respectively, and the 30-day readmission rate among them has been reported at 3.6% for total knee arthroplasty and 4.6% for total hip arthroplasty [13,14]. Notably, further statistical analyses need to be conducted to determine whether differences in terms of other complications are significant. Our results demonstrate that patients with severe postoperative anemia had overall higher rates of blood transfusions, readmissions, reintubations, and complications. As an elective procedure, healthcare providers must weigh the risks and benefits of TJAs when offering these interventions to their patients, and keep patients’ specific health conditions in mind when addressing risks and complications.

Patients who had preoperative anemia comprised 14.8% of elective knee joint arthroplasty recipients and 22.9% of those who underwent elective hip joint arthroplasty in one study [15]. In addition, the incidence of preoperative anemia was almost twice as high in patients aged 80 years and older compared to those younger than 80 years, indicating substantially higher rates of transfusion among older patients. In anemic patients, analyses have identified mild anemia (hematocrit of 27-36%) as a significant risk factor for mortality, renal complications, respiratory complications, sepsis, wound infection, and urinary tract infections [15]. Severe anemia (hematocrit <27%) was also a risk factor, with a higher odds ratio for total complications. Both mild and severe anemia were significant risk factors for increased rates of perioperative blood transfusion, non-home discharge, and unplanned hospital readmissions. In addition, it has been shown that postoperative infections and cancer recurrence may occur more often in transfused patients, especially if given after cardiac, abdominal, and orthopedic surgeries [16]. Evidence also demonstrates that dose-related augmentation of postoperative infections is correlated with blood transfusions [17]. These infections can result in longer intensive care unit stays or hospital courses and increased hospital costs. Given the risks associated with transfusions, interventions to limit the need for transfusions in this patient population would improve long-term outcomes and reduce hospital expenditure.

A possible way to limit transfusions and prevent the risks associated with transfusion administration is to consider the type of anesthetic used in this patient population. In this study, the incidence of postoperative transfusions was significantly lower in anemic patients who received neuraxial vs. general anesthesia, and this may point towards advantages driven by anesthesia type. Neuraxial anesthesia has been associated with decreased postoperative ventilator use, cardiac arrest, stroke, and unplanned intubations [18]. Patients receiving spinal anesthesia also demonstrated a lower rate of wound infection, blood transfusions, and overall complications. The length of surgery and hospital length of stay were both decreased in the spinal anesthesia population. These effects were more pronounced among patients with numerous comorbidities. However, there are potential complications with this approach as well, such as spinal hematoma, epidural abscess, and nerve injury [19]. With proper technique, these adverse outcomes can be avoided while providing the benefits of neuraxial anesthesia.

This study has a few limitations, especially driven by its retrospective study design. Rather than concluding that severe anemia causes perioperative complications, or that neuraxial anesthesia directly minimizes these complications, we have found associations between these factors and outcomes. Future prospective studies can further elucidate the strength of the associations we have identified here. Although NSQIP is a comprehensive registry, some important factors such as surgical settings (e.g., ambulatory surgery center, academic hospital, etc.) were not present in the database. In parallel, sites participating in NSQIP tend to be large academic centers, thus creating datasets under-representative of community settings where TJA may occur. Lastly, variable coding for outcomes such as hospital readmission was not accompanied by the reason for admission, limiting our view regarding the link between anemia and readmission.

## Conclusions

TJA procedures such as hip and knee arthroplasties are elective surgeries that can potentially provide substantial pain relief and improve the quality of life in patients who need surgical intervention. However, not all patients are the best-suited candidates for this therapy, as postoperative readmissions, complications, and transfusions represent risk factors for all TJA patients. More vulnerable populations such as severely anemic patients are at greater risk of postoperative blood transfusions, and this study provides insight into how healthcare providers can better prevent adverse effects in this population. Neuraxial anesthesia can reduce the risk of postoperative transfusion in severely anemic patients undergoing TJAs, ultimately leading to reduced discomfort, hospital expenditure, and adverse outcomes.
